# The Challenges and Prospects of p53-Based Therapies in Ovarian Cancer

**DOI:** 10.3390/biom13010159

**Published:** 2023-01-12

**Authors:** Bryce Wallis, Katherine Redd Bowman, Phong Lu, Carol S. Lim

**Affiliations:** Department of Molecular Pharmaceutics, College of Pharmacy, University of Utah, Salt Lake City, UT 84112, USA

**Keywords:** ovarian cancer, gene therapy, p53 therapies, HGSOC, gene delivery

## Abstract

It has been well established that mutations in the tumor suppressor gene, p53, occur readily in a vast majority of cancer tumors, including ovarian cancer. Typically diagnosed in stages three or four, ovarian cancer is the fifth leading cause of death in women, despite accounting for only 2.5% of all female malignancies. The overall 5-year survival rate for ovarian cancer is around 47%; however, this drops to an abysmal 29% for the most common type of ovarian cancer, high-grade serous ovarian carcinoma (HGSOC). HGSOC has upwards of 96% of cases expressing mutations in p53. Therefore, wild-type (WT) p53 and p53-based therapies have been explored as treatment options via a plethora of drug delivery vehicles including nanoparticles, viruses, polymers, and liposomes. However, previous p53 therapeutics have faced many challenges, which have resulted in their limited translational success to date. This review highlights a selection of these historical p53-targeted therapeutics for ovarian cancer, why they failed, and what the future could hold for a new generation of this class of therapies.

## 1. Introduction

Advancements in treatment options for ovarian cancer patients have resulted in a steady rise in the overall 5-year survival rate of these patients to around 47% [1]. However, treatment of the most common subtype of ovarian cancer, high-grade serous ovarian carcinoma (HGSOC), has thwarted scientists and clinicians alike, with the 5-year survival rate hovering around 29% for the last 30 years [2,3,4,5]. Ovarian cancer kills more women than any other gynecological cancer, surpassing breast cancer deaths, and is the fifth leading cause of death in women, despite accounting for only 2.5% of all female malignancies [1,6,7,8]. The high mortality rate is typically attributed to late-stage diagnosis (75% of patients diagnosed at stage three or four) in which cancer cells have spread outside of the pelvic cavity [7,9]. Late-stage diagnosis is associated with the lack of accurate diagnostic tools currently available as well as vague symptoms and asymptomatic cases [6,10,11]. The symptoms of ovarian cancer—abdominal fullness, bloating, abdominal pain, and gastrointestinal symptoms—are often misinterpreted as menopause or other diseases, delaying diagnosis [6,11]. Furthermore, the tissue origin of ovarian cancer has often been debated, adding to the lack of adequate detection methodologies and obstacles for discovering treatments [12].

## 2. Ovarian Cancer Types

Ovarian cancer, defined as cancer of the ovaries and/or fallopian tubes, is classified via histological subtypes: epithelial (~80–95%), germ cell (~15%), and sex-cord stromal (~5%) [10,12]. The most prevalent histological type, epithelial ovarian cancer (EOC), is further categorized into serous (~50%; low-grade or high-grade), mucinous (~5%), endometrioid (~15%), and clear cell (~5%) [10,12]. In clinical settings, low-grade (LGSOC) and high-grade (HGSOC) serous ovarian carcinomas are typically differentiated based on their mutation profiles [13]. LGSOC (Type I) contains mutations in the mitogen-activated protein kinase (MAPK) pathway—KRAS, BRAF, or ERBB2 genes—and, importantly, does not possess any p53 mutations [13,14]. Contrarily, HGSOC (Type II) is characterized by p53 mutations but has no mutations in the MAPK pathway [13,15]. Patients with LGSOC tumors have slower progressing disease and better survival outcomes compared to those with HGSOC [13]. Advanced-stage HGSOC accounts for the most ovarian cancer diagnoses (~50%) and ovarian cancer deaths (~70%) [10,12,16].

## 3. Origin of Ovarian Cancer

The diagnosis and detection of HGSOC are challenging partly because classification and subsequential treatment/response rates of ovarian cancer types are still controversial. Historically, two hypotheses of the origin of HGSOC have prevailed: origination in the fallopian tube epithelium, or in the ovarian surface epithelium. One hypothesis is that HGSOC originates in the fallopian tube at the oviductal fimbriae due to the expression of p53 in ovarian inclusion cysts and fallopian tube fimbriae [2,17]. On the other hand, HGSOC has also been postulated to originate from mutations resulting from normal ovulatory wounds in the ovarian surface epithelium [17,18]. More recent studies suggest that HGSOC can originate at both the ovarian surface epithelium and the fallopian tube fimbriae [18,19,20]. Scientists are exploring biomarker candidates to differentiate between the origins of HGSOC, including SOX18, a transcription factor associated with fallopian-derived HGSOC [20]. Understanding the genetic landscape and biomarkers of HGSOC is critical to take strides towards developing better treatment options for patients.

## 4. Biology and Genetic Landscape of Ovarian Cancer

The heterogeneous genetic and epigenetic nature of EOC has further thwarted researchers and created hurdles for developing novel treatments. Interestingly, EOC continues to be categorized as a single disease even though research has shown that the biological and genetic backgrounds of this group of tumors is highly variable [2]. The grade system that was introduced for LGSOC and HGSOC has helped to distinguish between the mutational landscapes of EOC, but treatment guidelines for these very different diseases continue to be relatively similar [21]. Additionally, the lack of uniformity of genetic alterations has contributed to late-stage diagnosis due to an absence of biomarkers. Detection of ovarian cancer has relied on CA125 (Cancer Antigen 125, a mucinous glycoprotein found on the surface of ovarian cancer cells) and transvaginal sonography. CA125, the most well-established ovarian cancer biomarker, is known to be elevated in 50% of early-stage and 92% of advanced-stage ovarian cancers but has sensitivity issues, especially in the early stage of the disease [22]. The low incidence of ovarian cancer also means that routine CA125 screening for healthy women results in a high false positive rate: about 80% of women who show elevated CA125 levels do not have ovarian cancer [23]. Recent studies have identified potential HGSOC biomarkers FGA, VWF, ARHGDIB, and SERPINF2 [24], which may be useful for HGSOC detection in the future.

Genetic alterations have been more defined compared to biomarkers in ovarian cancer and include BRCA1, BRCA2, p53, KRAS, BRAF, PIK3CA, HOXA11, and PTEN [2,7,25]. HGSOC is predominantly characterized by mutations in p53, which occur in up to 96% of HGSOC patients, with some population variability [16,26]. The genetic predispositions of defective BRCA1 and/or BRCA2 (the second most common mutations in ovarian cancer) genes are responsible for only 10–20% of all ovarian cancer cases, with the genetic marker increasing a patient’s risk by 15–60% [7,27]. However, many of the currently available treatment options, such as PARP inhibitors veliparib and olaparib, are targeted agents for patients with BRCA1 and/or BRCA2 mutations and are therefore ineffective for many ovarian cancer patients [28,29].

## 5. Current Standard of Care for Ovarian Cancer

The current standard of care for ovarian cancer includes cytoreductive surgery, chemotherapy, bevacizumab (Avastin), and PARP (poly-ADP-ribose polymerase) inhibitors [28,29,30]. Non-specific chemotherapies including paclitaxel, cyclophosphamide, and platinum compounds are commonly used [31]. Anti-angiogenic agents, such as the monoclonal IgG1 antibody bevacizumab, have shown some improvement in progression-free survival; however, there are concerns about significant adverse effects in patients with advanced disease [31]. Additionally, studies indicate that only 16–21% of recurrent ovarian cancer patients show a response rate to bevacizumab [30]. Unfortunately, around 70% of ovarian cancer patients will relapse within three years following the traditional methods of surgery and platinum-based chemotherapy [32]. Therefore, targeted therapies have been an attractive approach for treating ovarian cancer.

Approved targeted therapies for ovarian cancer have predominantly been PARP inhibitors, which include veliparib, olaparib, rucaparib, and niraparib [33,34]. PARP enzymes are responsible for repairing single-strand DNA breaks via the base excision repair pathway [33]. BRCA1 and BRCA2 genes are responsible for repairing DNA double-stranded breaks via the homologous recombination pathway. PARP inhibitors are thus designed to target cancer cells with BRCA mutations and/or defective homologous recombination pathways. With both DNA repair mechanisms inhibited, the cancer cells die, while normal cells remain relatively unaffected [33,34]. PARP inhibitors are somewhat effective on HGSOC patients with germline or somatic BRCA mutations with defects in the homologous recombination in cancer cells. However, only 15–17% of HGSOC patients are reported to have germline BRCA mutations, and only 6% are reported to have somatic BRCA mutations, showcasing that PARP inhibitors can only be used on a small population of HGSOC patients [35]. Furthermore, it has been suggested that 40–70% of BRCA-mutated ovarian cancers do not respond to the PARP inhibitor treatment [33]. Additionally, olaparib has been linked to an increased risk of secondary cancers, due to the effect on the DNA repair pathway in healthy cells [32]. Overall, novel targeted therapies for HGSOC are gravely needed, and particularly therapies that serve a larger population of patients with varying disease etiology.

## 6. p53 and Cancer

p53 is the most mutated gene across most cancers (~50%) and can lose function or gain oncogenic activity when mutated [36]. Loss-of-function p53 tumor suppressor mutations can result in loss of cell cycle arrest, loss of apoptosis, loss of senescent growth arrest, chromosomal instability, and inefficient DNA base excision repair [37,38]. These alterations allow for the evasion of critical cell cycle mechanisms, thereby supporting cancer proliferation. On the other hand, gain-of-function p53 oncogenic mutations can result in accelerated tumor metastasis, altered transcriptional activation of target genes, inhibition of apoptosis, and enhanced resistance to chemotherapy [37]. Mutations in p53 have been shown to occur in upwards of 96% of HGSOC patients with both gain-of-function (oncogenic) and loss-of-function (loss of p53 activity) mutations occurring [6,10,16,39].

## 7. p53 Structure and Dysfunction in HGSOC

**Figure 1:**
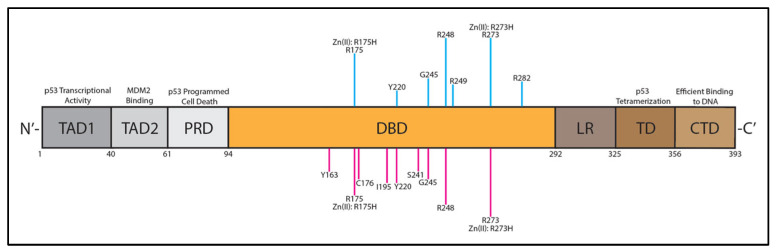
p53 domains (boxes) and cancer-inducing p53 mutations (pink and blue lines). Numbers below domains represent the amino acid number (1–393). Blue lines above DBD represent approximate abundances of all types of cancer-inducing p53 mutations. Pink lines below DBD represent approximate abundances of HGSOC-inducing p53 mutations. Zn(II) binding is inhibited by R175H and R273H mutations. TAD1 and TAD2 are p53 transcriptional activation domains. TAD2 is the MDM2-binding site. PRD (PXXP residues) is associated with p53 programmed cell death. CTD is highly unstructured and assists with efficient binding of p53 to DNA [45,46,49,52,53]. TAD1, TAD2 = transactivation domains 1 and 2; PRD = proline rich domain; DBD = DNA-binding domain; LR = linker region; TD = tetramerization domain; CTD = C-terminal domain.

Typically, p53 exists as a homotetramer (4 monomers that form a dimer of dimers) when acting at the nucleus in its primary role as a transcription factor, upregulating or downregulating numerous genes [40]. p53 also exists in monomeric or homodimeric form in the cytoplasm and at the mitochondria, regulating apoptosis as well as the autophagy process [41]. Recent literature has suggested that mitochondrial p53 interacts with anti-apoptotic factor Bcl-XL and apoptotic regulator Bak in dimeric and monomeric forms [42,43,44]. The domain structure of p53 consists of 393 amino acid residues with an N-terminal ubiquitin ligase (MDM2) binding site, amino-terminal transactivation domains TAD1 and TAD2, a proline-rich domain (PRD), a DNA-binding domain (DBD), a linker region (LR), a tetramerization domain (TD), and a carboxyl terminal domain (CTD), as shown in Figure 1. The CTD contains 3 nuclear localization signals that confer localization of p53 to the nucleus [45,46]. Sixty-two percent of all cancer-inducing p53 mutations (eighty-two percent for HGSOC) occur as missense mutations in the DBD (R175, Y220, G245, R248, R249, R273, or R282), resulting in the eradication of DNA-binding functions, either directly or indirectly; see Figure 1 (blue lines) [41,45,47,48]. A recent study by Tuna et al. highlighted the nine most common p53 hotspot mutations in the DBD specific to HGSOC (R273 = 20.63%, R248 = 16.67%, R175 = 14.29%, Y220 = 9.52%, I195 = 9.52%, C176 = 8.73%, G245 = 8.73%, S241 = 6.35%, Y163 = 6.35%); see Figure 1 (pink lines) [49]. Studies have shown that arginine 273 (R273) in the DBD is the most commonly mutated amino acid in p53-associated cancers, with 46.6% of mutations to histidine (R273H) and 39.1% mutations to cysteine (R273C) [50]. It has been suggested that these mutations in the DBD result in a structural change that alters the stability of p53 and becomes more rigid compared to wild-type (WT) p53 [50,51]. These key mutations in the DBD affect the function of p53, including apoptotic regulation, thereby allowing cancerous cells to proliferate unchecked.

p53 plays a pivotal role in maintaining homeostasis in cells by interacting with hundreds of genes when acting as a transcription factor [54,55]. The p53 function can become inactivated directly via mutation or indirectly via aggregation or regulatory proteins [51]. Mutation(s) in p53 can therefore have numerous outcomes, but typically result in the loss of WT p53 function [48]. Normally, p53 is kept at low levels via the E3 ubiquitin ligase MDM2 [47]. In response to stress such as DNA damage, p53 becomes upregulated and acts as a tumor suppressor via cell cycle inhibition, apoptosis, senescence, DNA repair, and autophagy [47,56]. When p53 is mutated and thereby inactivated in cancer cells, it can no longer respond to stress, and tumor proliferation occurs due to the absence of apoptosis. It has been suggested that p53 mutations and degradation of WT p53 through negative regulatory proteins such as MDM2 or MDM4 are two main mechanisms responsible for p53 inactivation [46]. MDM2 and MDM4 are ubiquitin ligases that help to target p53 for proteasomal degradation [46,57]. In lieu of mutation, p53 can be downregulated in cancers by the upregulation of MDM2 and MDM4 inhibitors [57]. Therefore, therapeutic approaches for non-mutated p53 cancers have focused on developing MDM2 and MDM4 antagonists [57]. Another important controlling mechanism of p53 regulation is the PI3K (phosphatidylinositol 3-kinase) pathway, which is overexpressed in roughly 40–70% of all ovarian cancer tumors [56,58]. In vivo and in vitro inhibition of PI3K resulted in regression in ovarian tumors [59]. Additionally, the loss of PTEN, which is a negative regulator of PI3K, is commonly found in ovarian cancer tumors [58]. Furthermore, common p53 mutations can result in protein aggregation due to exposure of a “sticky” sequence [51]. Recently, it has been suggested that p53 can aggregate in the cytosol in HGSOC, specifically cells with a p53 R248Q mutation, further affecting its functionality [51]. These separate and interconnected pathways illustrate the complexities regarding the regulation of p53, with many mechanisms still not fully understood. Taken together, the p53 pathway dysfunction in cancer cells leads to unchecked growth. It is no wonder why cancer cells have evolved to readily mutate and inactivate p53, “the guardian of the genome”, to evade tumor suppression and promote proliferation.

**Figure 2:**
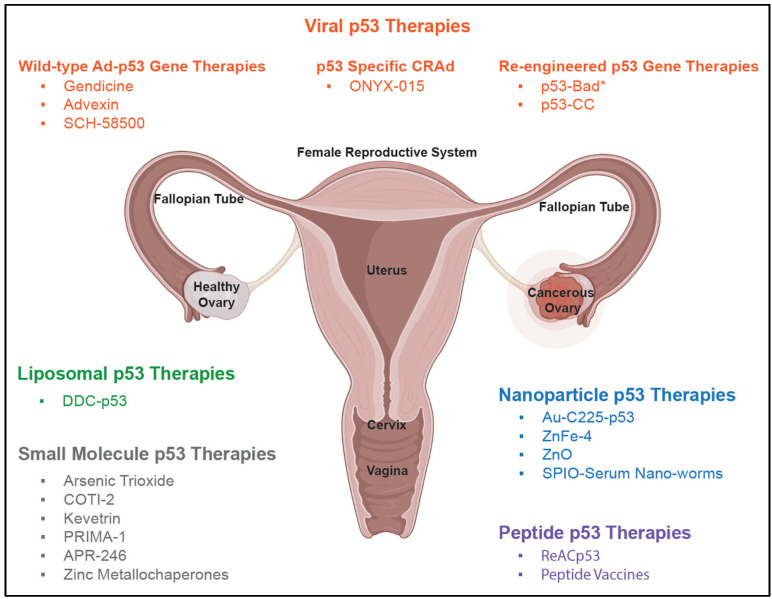
A selection of ovarian cancer p53-based therapies categorized into viral p53 therapies (wild-type Ad-p53 gene therapies, p53-specific CRAd, and re-engineered p53 gene therapies), nanoparticle p53 therapies, liposomal p53 therapies, peptide p53 therapies, and small molecule p53 therapies. Female reproductive system diagram from Bio Render.

Based on the nearly universal occurrence of p53 mutations in HGSOC, it is surprising that there are currently no FDA-approved p53-based therapies for HGSOC. However, there is a long history of research that has focused on WT p53, and p53-based therapies delivered via drug vehicles such as nanoparticles, viruses, polymers, and liposomes (see Figure 2) [60,61,62,63,64,65,66,67,68,69,70,71,72,73,74,75,76,77,78,79,80]. Interestingly, these creative therapeutics have yet to come to fruition. The following is a discussion of a selection of historical p53-targeted therapeutics for ovarian cancer (specifically for HGSOC), why they failed, and what the future could hold for newer p53 and p53-based therapies.

## 8. Early p53-Based Therapies

Due to the commonly mutated p53 gene, scientists have long desired to deliver WT p53 to rescue cancer cells with these mutations. It was hypothesized that the delivery of WT p53 to cancer cells would restore the functionality of p53 and thereby suppress tumor growth. A main obstacle for WT p53 gene therapy, as with many therapies, is the delivery system. Both viral and non-viral systems can be utilized as gene delivery vehicles. A comprehensive review of gene therapies by Drakopoulou et al. highlights the delivery systems that have been employed for gynecological disorders [81]. Viral systems include retroviral vectors, lentiviral vectors, adenoviral vectors (Ad), adeno-associated vector (AAV), oncolytic virotherapies, measles virus, and herpes simplex virus [81]. Non-viral systems include polymeric nanoparticles, lipid-based nanoparticles, inorganic nanoparticles, naked DNA plasmids, and liposomes [81]. Viral vectors have historically been the delivery vehicles of choice for WT p53 gene therapy, given the advantages of high gene transfer efficiency, large gene carrying capacity, selective gene delivery, mild cytotoxicity, potential therapeutic immunogenicity, ease of construction and manipulation, and cost-effective commercial manufacturing [82]. It is therefore logical that one of the earliest attempted p53-therapies was adenovirally delivered WT p53 (Ad-p53). Other early (prior to 2005) p53-targeted therapies reviewed here include liposomal p53 and p53-targeted oncolytic adenovirus. A summary of all p53-based therapies is found in Figure 2.

### 8.1. Wild-Type Ad-p53 Therapies: Gendicine, Advexin, and SCH-58500

In 2003, the first ever gene therapy, Gendicine (recombinant WT p53 Ad5), was approved in China for the treatment of head and neck cancer [82,83]. Ad-p53 therapies demonstrated promising safety and efficacy profiles for the treatment of head and neck squamous cell carcinoma [83,84]. Due to the initial success, Ad-p53 therapies gained a surplus of attention, and by 2005, 58 recombinant Ad-p53 therapies had reached trials for over 20 types of cancers, including Advexin and SCH-58500 for ovarian cancer [85,86,87]. Gendicine, Advexin, and SCH-5800 consist of human WT p53 delivered via a recombinant Ad5 that has a deleted E1B region (responsible for viral protein synthesis) [87,88]. Ad5 was chosen due to its weak pathogenicity characteristics compared to other adenoviruses [86]. Gendicine, Advexin, and SCH-58500 are replication-deficient adenoviruses, thereby ensuring that they cannot reproduce within target cells [85,87,88].

**Gendicine**: To date, Gendicine is the only WT p53-Ad that has been approved in the world. Phase I, II, and III clinical trials were performed for advanced head and neck squamous cell carcinoma, while other studies were performed on advanced lung and liver cancers [86]. Initially approved in China for head and neck cancers, Gendicine has been used off-label for ovarian cancer and shown mixed results, with the earliest trials showing complete failure and more recent (2009/2010) preclinical studies suggesting a response rate of 90% in ovarian cancer patients [82,89]. With a decade since the last published update, the status of Gendicine in ovarian cancer in China remains unclear. Due to the contradictory translational implications, Gendicine has not been approved in the United States for the treatment of any type of cancer.

**Advexin**: A 2003 phase III clinical trial for Advexin (INGN 101; Ad5CMV-p53) was performed for head and neck cancer, while a 2003 phase II clinical trial was performed for non-small lung cancer [90]. In 2003, it was also indicated that Advexin would undergo clinical trials for lung, breast, ovarian, liver, and brain cancers [90]. A phase I clinical trial for the intraperitoneal delivery of Advexin to patients with platinum or paclitaxel-resistant EOC concluded that Advexin was well tolerated by patients, and 24% of treated patients had a stable disease during treatment [91]. With the lack of disease regression due to Advexin, the authors recommended further refinement of the gene therapy before starting additional trials, suggesting that WT p53 gene therapy alone may not be an adequate treatment for EOC [91].

**SCH-58500**: SCH-58500 progressed further in clinical trials for ovarian cancer compared to Advexin. Phase I and II clinical trials were performed on patients with recurrent ovarian, primary peritoneal, or fallopian tube cancers with mutated p53 [60]. The authors concluded that intraperitoneal delivery of SCH-58500 was safe and well tolerated, and when combined with platinum-based chemotherapy it was found to be associated with CA125 reduction in the patient types mentioned above [60]. Following these initial trials, a long-term study was performed to evaluate the survival rates of patients. This study found that the 12-to-13-month median survival of pretreated patients with recurrent ovarian cancer compared favorably to the 16-month median survival for those treated with paclitaxel (at the time of initial recurrence). This was an improvement, which implied a clear indication for the further study of SCH-58500 [61]; however, further studies of SCH-58500 for ovarian cancer following 2003 were not published to our knowledge.

### 8.2. Liposomal WT p53 Therapies

Liposomal drug delivery systems have been in mainstream use due to their reduction in drug toxicity and increased accumulation at target sites [92]. Liposomal delivery of WT p53 is an alternative non-viral gene delivery system that, while possessing lower transfection efficiency than viral vectors, is less immunogenic and easier to control [70]. Due to the challenges of viral WT p53 mentioned above, alternative delivery systems such as liposomes were developed. In an older study, a cationic liposome (DDC) composed of dioleoyltrimethylamino propane (DOTAP), 1,2-dioeoyl-3-phosphophatidylethanolamine (DOPE), and cholesterol was formulated to deliver WT p53 DNA to OVCAR-3 ovarian cancer cells and nude mice [70]. This study was based on the preliminary proof-of-concept findings from a 2001 phase I clinical trial for ovarian and breast cancer patients delivering the E1A gene via cationic liposome DC-Chol (DCC-E1A) [93]. The results of the DDC-p53 study indicated that the apoptotic p53 pathway was restored following treatment with DDC-p53 in vitro and in vivo and presented a novel liposomal delivery vehicle for WT p53 [70]. However, there are no recent follow-up studies nor clinical trials on DDC-p53 for ovarian cancer. Other cationic liposome delivery systems exist, such as PEGylated DC-Chol/DOPE cationic liposomes for the delivery of siRNA loaded against the kinesin spindle protein for ovarian cancer, but none for the delivery of p53 [94].

### 8.3. p53-Specific CRAd: ONYX-015

One of the first re-engineered adenoviral therapies that targeted mutant p53 was ONYX-015, which was designed as an oncolytic, conditionally replicative adenoviral vector (CRAd) for tumor-selective replication [87,95,96]. ONYX-015 (d1l1520 or CI-1042), a chimeric Ad, is comprised of type 2 and type 5 Ad with the deletion of part of the protein synthesis E1B-55 kDa-encoding region [87]. This CRAd is engineered to replicate only in cells with inactive p53, and thus selectively targets p53-deficient cancer cells for replication and leaves cells with unmutated p53 unaffected [97]. However, there were conflicting results around ONYX-015’s ability to differentiate between p53 mutated and non-mutated cells [87,98,99]. A phase II clinical trial was performed on patients with recurrent head and neck cancer p53 mutations. The results indicated that the surrounding healthy tissues were successfully not infected by CRAd; however, only 21% of patients had significant tumor regression [100]. Furthermore, when tested for metastatic colorectal cancer, a large number of patients were withdrawn from the study due to cancer progression [100,101]. A phase I trial of ONYX-015 was attempted for ovarian cancer and found no clinical response in patients [102]. Due to the limited tumor regression in clinical trials, ONYX-015 has not been approved for ovarian cancer treatment. Other replication competent CRAd vectors expressing WT p53 that have been tested in a variety of cancers include H101, AdΔ24-p53, SG600-p53, and OBP-702 [85,103].

## 9. The Failure of Early p53-Based Therapies in HGSOC

**Figure 3:**
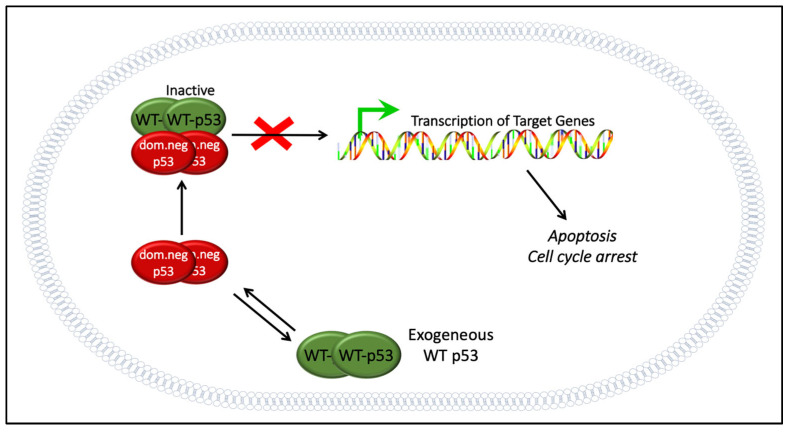
Why wild-type (WT) p53 gene therapy is not effective: dominant negative effect. Endogenous mutant p53 (dominant negative) found in cancer cells can tetramerize and inactivate any exogenously added WT p53. This inactive heterotetramer cannot bind to, nor activate, target genes. The result is that WT p53 cannot execute apoptosis nor cell cycle arrest. Bypassing the dominant negative effect may be possible with re-engineered p53 chimeras that do not bind to dominant negative p53 [52,104,105,106].

There are two major obstacles facing p53-based gene therapy that need to be addressed before it can become a reality. The first lies within the nature of mutant p53 found in cancer cells, where endogenous mutant p53 exerts a dominant negative inhibition of WT p53, reducing or eliminating the tumor-suppressing activities of WT p53 (Figure 3). The second major obstacle for p53-based gene therapy stems from the limitation of the delivery systems.

### 9.1. The Dominant Negative Effect

WT p53-based gene therapy has been lagging in the United States, despite the use of Gendicine for head and neck cancer in China for more than a decade [82]. When used for head and neck cancer treatment, Gendicine had a higher complete and partial response rate when used in combination with the standard treatment regimen compared to the standard regimen alone [82]. Since head and neck cancer cancers are highly heterogenous, especially between tumors at different anatomical sites, the p53 gene mutation frequency is also widely different, ranging from 30% to 70% [107]. Therefore, the extent of p53 mutations and the dominant negative effect on the efficacy of Gendicine in head and neck cancer are unclear. However, this may be a different story for HGSOC, where p53 gene mutation and deletion account for more than 96% of all cases [15].

WT p53 gene therapy for ovarian cancer has been attempted in the U.S. before [60]. Despite some promising preclinical studies [108,109], WT p53 gene therapy failed in clinical trials for ovarian cancer, as discussed extensively by Zeimet and Marth [5]. Unsurprisingly, the dominant negative effect was listed as one of the top challenges. In a group of cancer cells with heterogenous p53 genotypes, cells with WT p53 were susceptible to WT p53 therapy, but not cells expressing mutant p53 [4]. This supports the idea that the dominant negative effect may have played a large role in the failure of WT p53 therapy in ovarian cancer (Figure 3). The conflicting results from preclinical studies—which seemed capable of overcoming the dominant negative effect—and clinical trials may be explained by the extent of mutant p53 expression and how successfully p53 gene therapy can be delivered. When a strong promoter such as CMV is used to drive the expression of WT p53, it is possible that the endogenous mutant p53 could be oversaturated with WT p53, and some degree of activity may still be observed [54,55,105]. Nevertheless, this observation may be hard to achieve in clinical trials, especially if intratumoral injection is not available and delivery is insufficient.

### 9.2. Delivery Challenges

In addition to the failure of WT p53 delivered in vivo to overcome the dominant negative effect, differences in delivery methods in subcutaneous models [5,108,109] may confound results. While intratumoral injections in subcutaneous models may have succeeded, an in vivo study with adenovirus-carrying WT p53 delivered intraperitoneally for ovarian cancer with p53 mutation failed to produce a significant difference in the survival outcome [110]. Obviously, models using the intratumoral injection of subcutaneous tumors vs. intraperitoneal delivery are very different, with likely reduced Ad-p53 viral loads in the latter.

The delivery vector also affects the success of a therapeutic method, and the WT p53 gene therapy is no different. Severe liver toxicity and immunogenicity limit the use of the systemic delivery of naked adenovirus [111]. One feature of HGSOC that makes it attractive for viral vector delivery is the possibility of intraperitoneal (IP) delivery, which can avoid systemic toxicity and permit higher viral doses in theory [5]. Unfortunately, another obstacle expected to adversely affect the outcome of adenoviral p53 gene therapy is the prevalence of neutralizing antibodies against adenovirus, which are a concern for all adenoviral gene therapies [112]. The infectivity of adenovirus is known to be greatly inhibited by neutralizing antibodies presented in the ascitic fluid from ovarian cancer patients [113].

Neutralizing antibodies against Ad5, the most common serotype for transgene delivery, are present in 37% of the population in the United States, and the prevalence of neutralizing antibodies is even higher in many countries [114]. Pre-existing immunity against Ad5 is a challenge in cancer gene therapy (and other diseases as well). Preloading Ad fiber protein or pseudotyping can bypass the inhibitory effect of the neutralizing antibodies to allow better transduction efficiency in the short term [112], but this runs the risk of more severe immunogenicity and higher antibody titers in the long run. Nevertheless, it should be noted that adenoviral delivery has been utilized for COVID-19 vaccines, making the mainstream use of adenovirus a reality [115]. At the same time, p53-based cancer gene therapy may require higher transduction and gene expression efficiency than vaccines because it likely requires the majority of the cancer cells to express the therapeutic p53 to be effective.

The adeno-associated virus (AAV) is a popular alternative delivery vector. AAV has a lower packaging capacity, variable protein expression levels between serotypes, and the potential for random integration into the host genome, but has the potential of long lasting gene expression and a good safety record [116]. AAV has been reported to trigger low levels of immune responses, but would likely also face the same problem with neutralizing antibodies [117]. AAV type 2 antibodies are found in the ascitic fluid of more than 70% of ovarian cancer patients, indicating the possible limitation of AAV as an alternative vector [117].

Another challenge for adenovirus delivery is the lack of coxsackievirus-adenovirus receptor (CAR) in ovarian cancers [5,111,118]. The presence and prevalence of CAR is correlated to the transduction efficiency of the adenovirus since it is required for the homing and uptake of the virus to the host cells via endocytosis [119]. There are many ongoing studies to exploit alternative receptors such as folate or integrin receptors for increased viral uptake [120,121,122], with some ongoing studies in early clinical trials for glioma [123].

### 9.3. The Potential of WT p53 Gene Therapy

The effect of different p53 mutations on prognostic and treatment outcomes for HGSOC is a topic of debate. In the past, it has been argued that p53 mutation is not of substantial prognostic significance due to its almost universal presence in HGSOC [15]. However, different p53 hotspot mutations have remarkably different protein expression patterns, and each individual p53 mutation may have an impact on the outcome in patients with HGSOC [49]. Sensitivity to platinum compounds is also dependent on the p53 status in ovarian cancer [124]. However, the question that remains unanswered is if the re-introduction of p53 alone is enough for cancer regression in HGSOC.

Previously, WT p53 and cisplatin combination therapy failed to provide a long-term survival advantage [125]. However, the dominant negative effect and other limiting factors mentioned above could greatly affect the usefulness of WT p53 gene therapy in this combination study. The incorporation of an improved p53-based therapy (with an optimal delivery system) to a well-designed treatment regimen could still have promise as an effective strategy.

Advancements in gene delivery are also needed. Despite all the obstacles mentioned above, viral vectors are still the preferred delivery method of choice due to the higher transduction efficiency as compared to non-viral methods [126]. However, the controllable target moiety and non-immunogenic aspects of non-viral delivery methods have appeal as well. In recent years, the two delivery methods have been merged to achieve high transduction and low immunogenicity; these combined hybrid viral–non-viral methods may result in a safe and efficient p53-based gene therapy becoming a reality [120,126,127,128].

## 10. Next-Generation p53-Based Therapies

### 10.1. Next-Generation Viral Therapies

While WT Ad-p53 therapies demonstrated initial promise, they failed to treat ovarian cancer or gain FDA approval [5]. Therefore, researchers went back to the drawing board to develop more sophisticated next-generation, adenovirus-based virotherapy delivery for ovarian cancer. The following discussion highlights many of the adenoviral-based therapies that have been utilized for ovarian cancer in both clinical and preclinical trials (see Table 1 and Table 2 in ref. [96]).

A majority of these therapies have been strategically designed to overcome some of the specific barriers that plague ovarian cancer drug delivery. These methods include conditional replication, enhancing infectivity, restricting replication, increasing potency, and overcoming physical barriers and immunosuppression [96]. While there has been extensive work on adenovirus-based virotherapy delivery for ovarian cancer, seldom have these systems been employed to deliver p53. Two examples of this non-p53-specific adenoviral re-engineering are dl922-947 and Ad5-Δ24-RGD [96,129,130]. These next-generation mutants of CRAd ONYX-15 were designed to improve the oncolytic efficiency of their predecessors by deleting the Rb protein-binding site on E1A in lieu of E1B [96]. In vitro studies indicated that dl922-947 had a higher induction of lysis compared to wild-type Ad5 and dl1520 (ONYX-015), while in vivo xenograft mouse studies illustrated increased survival when treated with an intraperitoneal injection of dl922-947 [129]. The RGD-targeted adenovirus, Ad5-Δ24-RGD, was designed to enhance tumor infectivity by specifically targeting ovarian cancer cells with the RGD (Arg-Gly-Asp) motif mutation [130]. The initial phase I clinical trial showed promise for this novel CRAd with future chemotherapy combination studies in development [130].

Re-engineered adenoviruses for the delivery of p53 remain an untapped field with many other strategies still to be investigated. Next-generation CRAds, along with the other strategies mentioned above and detailed in other reviews [81,89], hold great promise for the future, both with and without p53 gene therapy.

### 10.2. Re-Engineered p53 Gene Therapies: p53-MTS, p53-Bad*, and p53-CC

An alternative strategy to WT p53 gene therapy is re-engineered p53 therapy, including attaching mitochondrial targeting signals (MTS) to full length p53 and p53 subdomains such as the DNA-binding domain for the rapid (but transient) induction of apoptosis in cancer cells. This is a novel mechanism of apoptosis unrelated to the activation of p53 target genes in the nucleus [52,165]. A newer iteration of this is p53-Bad*, which was designed by our lab to intrinsically induce apoptosis at the mitochondria by fusing monomeric p53 to the proapoptotic factor, Bad, which contains an embedded MTS [104]. Both p53-MTS and p53-Bad* were engineered to overcome the dominant negative effect that contributed to the failure of conventional WT p53 therapy (see also Figure 3) [104]. For p53-Bad*, two serine residues on Bad were both mutated to alanine (S112A and S136A, designated as Bad*) to avoid phosphorylation and control the localization of Bad solely to the mitochondria [104]. This novel gene therapy showed enhanced apoptotic activity in vitro across a variety of p53-mutated ovarian cancer cell lines, indicating that it could be utilized as a treatment regardless of p53 status [104], and it more recently showed tumor suppression in a zebrafish model of liver cancer [166].

Other re-engineered p53 gene therapies include “super p53” which has swapped out the tetramerization domain of p53 with an alternative coiled-coil tetramerization domain from B cell receptor (BCR), an intrinsic inducer of apoptosis, called p53-CC [167]. This re-engineered p53-CC avoids the dominant negative inhibition by mutant p53 found in cancer cells due to the inability to tetramerize with mutant p53 [168]. p53-CC can still tetramerize with itself and activate WT p53 target genes [54,55,167,168]. The major difference between p53-CC and the mitochondrially targeted p53 constructs (p53-MTS and p53-Bad*) is its ability to affect hundreds of p53 target genes, just as WT p53 does [167] for robust tumor suppression. To our knowledge, these are the only experimental re-engineered p53 therapies for ovarian cancer.

### 10.3. Nanoparticle p53 Therapies

**Au-C225-p53 Nanoparticles:** Given the failure of adenoviral WT p53 therapy in ovarian cancer, research has focused on developing more effective delivery systems to administer WT p53. A gold nanoparticle-based EGFR (epidermal growth factor receptor)-targeted system was developed for the delivery of WT p53 to ovarian cancer cells [73]. This delivery system was based on EGFR overexpression in many cancers, including in up to 90% of ovarian cancers [169], and utilized the FDA-approved monoclonal antibody, cetuximab (C225), to specifically target EGFR for the delivery of p53 to ovarian cancer cells [73]. A sophisticated gold nanoconjugate system (Au-C225-p53) containing gold nanoparticles, cetuximab, and pCMVp53 plasmid revealed promising results for targeting ovarian cancers in vitro (SKOV-3 cells) and in vivo (SKOV-3 xenograft mice) [73]. Previously, gold nanoparticles have shown potential due to their ability to be applied photothermally and as a radiotherapy [73]. Currently, gold nanoparticles are a new drug delivery system with potential due to their scalable fashion, functional diversity, control of particle size and surface, and ability to differentiate between cells via surface coatings [73]. While this study showed utility in xenograft mice, it is still early to predict if this drug delivery system will make it to clinical trials.

**ZnFe-4 Nanoparticles:** Another approach being explored is to degrade p53 tumor-promoting, gain-of-function mutations in cancer cells, thereby mitigating the dysfunctional properties of cancerous p53. One study synthesized zinc ferrate (ZnFe-4) nanoparticles that had high specificity for a mutated p53 via the induction of ubiquitination-mediated proteasomal degradation, while not affecting WT p53 [76]. This study was based on previous findings that suggested zinc curcumin complexes could restore the functionality of p53 with R175H and R273H mutations, as these mutations can result in a loss of Zn(II) that is typically bound to the WT p53 core and result in aggregation [170]. In the DNA-binding domain, p53 contains one Zn(II)-binding site (Figure 1) that is essential for functionality [76]. A potential strategy to restore p53 functionality in cancer cells that specifically result in the loss of Zn(II) binding could be to deliver Zn(II). The authors used magnetic ZnFe nanoparticles which allowed for an efficient MRI T2 contrast effect as a theranostic. They hypothesized that these ZnFe nanoparticles would be able to induce p53 degradation in gain-of-function, loss-of-Zn(II) cancer cells [76]. They tested their nanoparticle construct in vitro in p53 S241F ES-2 ovarian cancer cells and in vivo in an orthotopically implanted p53 Y220C patient-derived xenograft breast cancer model [76]. Their results showed that the ZnFe-4 nanoparticles degraded mutated p53 with minimal toxicity [76]. This is another potential use of nanoparticle technology that could be attempted against ovarian cancer with gain-of-function mutated p53.

**Zinc Oxide Nanoparticles:** Studies have suggested that ZnO nanoparticles can induce apoptosis in ovarian cancer SKOV3 cells via size-dependent cytotoxicity [75,131]. These nanoparticles killed ovarian cancer cells mainly independently of p53 mutation status (with the exception of p53 R273H-mutated ovarian cancer cells, which did not respond) in vitro while inducing a much smaller effect in normal mouse fibroblasts, suggesting their potential as a general ovarian cancer therapy [75,131]. Preliminary data suggest that this delivery vehicle appears to have a stronger translational capability compared to ZnFe-4 nanoparticles as it appears to target both WT and mutant p53, an important characteristic for a successful p53 therapy. However, this study has only been performed in vitro and the results have yet to be confirmed in vivo or in a clinical setting; the lack of efficacy in R273H mutants suggests that further research into the mechanism of action is needed.

**SPIO-Serum Nanoworms:** Iron oxide-based nanomaterials have been used to induce ferroptosis, an iron- and ROS-dependent cell death pathway. Superparamagnetic iron oxide nanoworms were incubated with human serum in combination with the overexpression of p53 to induce ferroptosis in ovarian cancer cell lines [171]. It is hypothesized that iron-based nanomaterials can be utilized to deliver iron to cancer cells and induce ferroptosis [171]. The induction of the iron-dependent cell death pathway, ferroptosis, could be an alternative mechanism for promoting cell death in lieu of apoptosis in overexpressed mutated p53 ovarian cancers. The authors provided experimental evidence that supported their hypothesis that the SPIO serum facilitated ferroptosis in vitro in two ovarian cancer lines, SKOV3 and A2780 [171], which made it challenging to draw any general conclusions based on the heterogeneity of ovarian cancer. Follow-up studies with more diverse cell lines with a variable p53 status are needed. Additionally, intracellular iron regulation in cancer environments remains poorly understood, making this a difficult strategy [171].

### 10.4. Peptide p53 Therapies

**ReACp53:** One common class of p53 mutations in cancer are structural mutants, such as R175H and R282W [133]. These mutations destabilize the structure of p53 and result in p53 aggregation, which leads to dominant negative and gain-of-function activity [133]. ReACp53, a 17-residue peptide designed to rescue the activity of p53, releases p53 from aggregation in primary ovarian cancer cell samples, restoring its function [79]. ReACp53 was shown to be effective against R175 and R248 p53 mutants, but not WT p53 [79]. Intraperitoneal ReACp53 significantly shrunk the tumor size in a mutant p53 ovarian cancer mouse xenograft model with minimal toxicity [79]. Further, combining ReACp53 with carboplatin treatment showed synergistic and/or additive effects in a subset of ovarian cancer cell lines and an Ovcar3 ovarian cancer mouse model, indicating that ReACp53 may be able to increase the efficacy of carboplatin [133].

**Peptide Vaccines:** The p53-SLP vaccine is a p53-targeted vaccine using overlapping synthetic long peptides to replicate the most immunogenic section of WT p53 (amino acids 70 to 248 in p53). It was developed to target cancers with overexpression of p53 which occurs in 50–60% of ovarian cancers, with about half of those associated with mutant, oncogenic—gain-of-function—p53 [134,135]. Administration of this p53-SLP vaccine was deemed safe and induced a p53-specific immune response, but a phase II clinical trial showed no change in response to secondary chemotherapy in immunized patients vs. historical data [134]. Pretreating ovarian cancer patients with cyclophosphamide before p53-SLP immunization was also attempted, and, while the study showed a strong p53 immune response, only 2 of the 10 patients involved in the study responded with a stable disease (no complete/partial response, no disease progression) after treatment [136]. Combining p53-SLP with INF-alpha and gemcitabine also increased the immune response to p53 in patients with platinum-resistant ovarian cancer [137].

A clinical trial of a second p53 vaccine using a short peptide (WT p53 amino acids 264–272) was tested in combination with low-dose IL-2 (interleukin 2) and administered either subcutaneously or by pulsing the peptide on dendritic cells. Both methods of administration resulted in a potent p53 immune response. Though some high-grade adverse events occurred, these were associated with IL-2 rather than the p53 vaccine [138]. Unfortunately, there was no significant difference in overall survival between the different arms of this study [138,172], and further studies in ovarian cancer have not been reported.

### 10.5. Small Molecule p53 Therapies

**Arsenic Trioxide:** Arsenic trioxide (ATO) is capable of re-activating a variety of misfolded p53 structural mutants (e.g., R175H, R273H, and R248Q) by binding to three cysteine residues (C124, C135, and C141) in p53, which stabilizes the structure and restores transcriptional activity [139]. ATO successfully induced apoptosis and inhibited cell growth in ovarian cancer cell lines, including paclitaxel-resistant cell lines [140]. This effect was compounded when ATO was combined with cisplatin [140]. ATO has also been shown to increase the efficacy of PARP inhibitors against ovarian cancer, including platinum-resistant and homologous recombination proficient lines [140,141,173]. A phase II clinical trial for ATO-based sequential combined chemotherapy in ovarian cancer patients was recently completed in China, with 33 patients demonstrating an overall response rate of 42% and a disease control rate of 85% [142]. Adverse myelosuppression was a major concern, as it was seen in 100% of patients, and 73% had mid- to high-grade levels [142]. Another clinical trial (NCT04489706) for ATO in ovarian and endometrial cancers was posted for recruitment in 2020 [174].

**COTI-2:** COTI-2 is a small molecule thiosemicarbazone that can reactivate misfolded mutant p53 by stabilizing the structure, thus restoring transcriptional and DNA-binding activity [143]. COTI-2 has activity against ovarian cancer cell lines [144]. A clinical trial for COTI-2 in ovarian and other cancers (NCT02433626) began in 2015, and, though an early phase I dose escalation study indicated COTI-2 is safe and well tolerated [144], the final results have not yet been released. Another group is examining the derivatives of COTI-2, and report increased resilience to the development of cancer resistance with a terminal N-disubstituted derivative called COTI-NMe_2_ [145,146]. COTI-NMe_2_ showed an 11-fold lower IC_50_ in human colorectal carcinoma cells compared with COTI-2 [145], and it could be interesting to see if this translates to ovarian cancer.

**Kevetrin:** Kevetrin (thioureidobutyronitrile) is a small molecule that induces cell cycle arrest and apoptosis in cancer cells through a multi-pronged mechanism that includes the activation and stabilization of p53 [147]. Kevetrin inhibited tumor growth and lengthened survival time in ovarian cancer mouse models [147]. A recent phase II clinical trial (NCT03042702) showed stable disease in ovarian cancer patients [175], but with only two enrolled patients; these results require replication if Kevetrin is to progress as an ovarian cancer treatment.

**PC14586:** PC14586 is a small molecule that binds to and reactivates p53 with Y220C mutations [46]. Because only 2% of cancers have Y220C p53 mutations [46], PC14586 is currently only being tested in a phase I/II clinical trial (NCT04585750) for patients with solid tumors with Y220C p53 mutations, including ovarian cancer patients [164]. Though no final results have been posted yet, preliminary studies indicate that PC14586 has a good safety profile [163], and with 9.5% of HGSOC expressing Y220C-mutated p53 [49], PC14586 certainly holds promise for this particular subset of patients.

**PRIMA-1 and APR-246:** PRIMA-1 (p53 reactivation with the induction of massive apoptosis) reactivates several p53 mutants through refolding, reduces mutant p53 aggregation in ovarian cancer cells [148], and can sensitize chemotherapy-resistant ovarian cancer cells to cisplatin [149]. PRIMA-1 has also been combined with SHetA2, a small molecule heteroarotinoid that induces G1 cycle arrest and apoptosis in cancer [150] and may also activate p53 by interfering with p53 repression by mortalin, a protein that can bind and inactivate p53 in the cytoplasm [151,176]. A phase I SHetA2 clinical trial (NCT04928508) in advanced/recurrent ovarian cancer is currently recruiting [177]. Together, PRIMA-1 and SHetA2 had additive effects and prevented ovarian cancer tumor growth in 67% of mice [32], indicating that this combination may be effective as a maintenance therapy for ovarian cancer patients.

APR-246 (PRIMA-1^MET^), a methylated analog of PRIMA-1, has largely replaced PRIMA-1 in recent years due to its increased efficacy [152], and has appeared in >10 clinical trials [153], including two recent trials in ovarian cancer. Initial results for the phase II clinical trial (NCT03268382) of APR-246/doxorubicin in platinum-resistant HGSOC showed a disease control rate (complete response + partial response + stable disease) of 70% over 18 months for the main arm of 4.5 g APR-246 administered by IV over 6 h with pegylated liposomal doxorubicin [178]. A phase II clinical trial (NCT02098343) for APR-246 with carboplatin combination chemotherapy in patients with recurrent HGSOC showed only 75% disease control for patients treated with the APR-246 and carboplatin combination compared to 85% disease control for patients treated with the carboplatin combination alone; however, patients in the APR-246/carboplatin arm had more complete responses compared to the carboplatin-only arm (9.5% vs. 2.8%) [179]. Cvrljevic et al. recently demonstrated that 22% of HGSOC tumors with lowered levels of CIP2A oncoprotein are particularly sensitive to APR-246 [154], suggesting that APR-246 therapy may be preferentially effective in CIP2A-deficient patient populations, which may warrant further trials in this population. Another possibility is combination treatment of APR-246 with mebendazole, an antiparasitic drug hypothesized to be effective against cancer due to its microtubule destabilization effects [155]. Mebendazole recently showed evidence of activity against ovarian cancer, including synergistic effects in ovarian cancer cell lines in combination with APR-246 [155].

**Zinc Metallochaperones:** Zinc metallochaperone (ZMC) compounds are a class of small molecules that restore zinc binding to reactivate mutant R175H p53, which has a reduced affinity for the zinc ion [157,158]. ZMC1 (also known as NSC319726) was identified through a drug screen and shown to successfully induce apoptosis in ovarian cancer cell lines, likely through a two-pronged mechanism of (1) restoring proper WT p53 folding through the delivery of high amounts of zinc ion, and (2) the activation of apoptosis through the ROS (reactive oxygen species) pathway [153,159]. Interestingly, ZMC1 had no synergistic effects with the tested chemotherapies (e.g., cisplatin) and radiation in ovarian cancer cells but did have synergistic efficacy with MDM2 and BCL2 antagonists [160]. Because ZMC-1 reactivates mutant p53 by drastically increasing zinc ion levels in cells [158], there is some concern that treatment with ZMC1 could have toxic off-target effects through zinc interactions with other factors [160]. Thus, some focus has been placed on exploring other ZMCs, such as ZMC2 and ZMC3, which have similar therapeutic profiles to ZMC1 [161] and C1/C85, and which the preliminarily results suggest are less toxic [162]. One study showed that cellular zinc homeostasis may be sufficient to control zinc toxicity; pharmacokinetic studies showed that ZMCs have a very short (<30 min) half-life, and cells can quickly normalize zinc levels, minimizing off-target effects [156]. This study also showed that a 30 min exposure of ZMC1 to ovarian cancer cells reduced colonies to about 50% of the control [156]. Overall, though ZMC compounds have not yet progressed to clinical trials, they show promise as a treatment for cancers with zinc-deficient p53 mutations.

## 11. The Future of p53-Based Therapies for HGSOC

The future remains bright for p53-based therapies (summarized in Figure 1 and Figure 2) for the treatment of HGSOC, given the high percentage of p53 mutations. However, there are several obstacles that need to be overcome for this class of therapeutics to be successful. Due to the abundance of heterogeneity associated with HGSOC, it is likely that one single therapy will not suffice for all patients. Therefore, treatment outcomes will depend on a variety of factors including, but not limited to, the following: specific p53 mutation status, BRCA 1/2 status, resistance to chemotherapy agents and/or PARP inhibitors, biomarkers, tumor origin site, stage, and Ad5-neutralizing antibody immunity status. Clinical therapies may need to be tailored based on a comprehensive analysis of these factors. Further research is desperately needed in this field to gain a greater understanding of ovarian cancer, including where it originates, how it spreads, and what biomarkers could be utilized for early detection.

Re-engineered p53 gene therapies and adenoviral vectors are promising up-and-coming tools in the field. Since WT p53 gene therapy has not been successful even in a variety of delivery vectors, re-engineered p53 gene therapies are an intuitive next step. For example, as mentioned previously, p53-Bad* has shown success at inducing apoptosis in ovarian cancer cell lines regardless of p53 status [104]. Re-engineered p53 therapies hold promise due to their potential to overcome the dominant negative effect and other barriers that have plagued WT p53 therapies. An ideal re-engineered p53 therapy would not discriminate based on p53 mutation status, and an ideal delivery vehicle would have a high transfection rate with minimal toxicity. Re-designed/improved vehicles that can circumnavigate the current barriers associated with delivery to ovarian cancer cells is one possible approach among many. Other novel promising p53-based therapies for FDA approval (outlined above) include Au-C225-p53, p53 vaccines, APR-246, and zinc metallochaperones.

There are many barriers facing p53-based therapies for the treatment of HGSOC, including but not limited to the heterogeneity of p53 mutations, delivery vehicles, a lack of diagnostic tools and biomarkers, non-p53 genetic mutations, chemotherapy resistance, and a general lack of understanding of the disease origin and progression. Re-engineered p53-based therapies tailored to the specific patient’s disease and delivered with improved vectors, in tandem with combination therapies when needed, hold great therapeutic potential for the treatment of HGSOC and ovarian cancer in general. Furthermore, combining p53 therapies with up-and-coming immunotherapies, such as adoptive T-cell transfer, oncolytic virus immunotherapy, cancer vaccines, and immune checkpoint inhibitors, are attractive approaches as they could target both the intrinsic and extrinsic apoptotic pathways, inducing cancer cell death from a multitude of angles [180,181]. The future of these therapies and further ovarian cancer research will dictate the prognosis for many patients and should thus be moved toward with urgency.

## Figures and Tables

**Table 1:** Selected p53-based therapies for ovarian cancer. Orange = viral vectors; blue = nanoparticles; green = liposomes; purple = peptides; gray = small molecules.

Name, Year, and Ref(s)	Gene Therapy, Small Molecule, or Peptide	Drug Delivery System	Target Population/ Mutation(s)	Function(s)	In Vitro, In Vivo, or Clinical Trial Phase	Response/ Outcome
**Gendicine** (2003) [5,82,84,85,86,87]	Gene Therapy	Ad5	p53 mutations	Restore WT p53 induction of apoptosis	Approved in China	Positive in head and neck cancer, failed in ovarian cancer
**Advexin** (2004) [85,87,91]	Gene Therapy	Ad5	Platinum and paclitaxel resistant; p53 mutations	Restore WT p53 induction of apoptosis	Phase I clinical trial	Well tolerated; dosing schedule and amount could not be deduced
**SCH-58500** (2002) [60,85,96]	Gene Therapy	Ad5CMV-p53 ΔE1A	p53 mutations	Restore WT p53 induction of apoptosis	Phase I and II clinical trialsNCT00002960	Well tolerated; Similar toxicity to traditional chemotherapy
**ONYX-015** (2002) [87,102]	NA	CRAd, dl1520 with E1B 55-kd gene deletion	p53 mutations; cisplatin-resistant	Restore WT p53 induction of apoptosis	In vivo p-53-deficient nude mouse xenograft; phase I; clinical development halted	Efficacy only satisfactory when used with other anticancer agents; discontinued in U.S.
**dl922-947** (2006) [129]	NA	CRAd dl922-947	Rb and p53 mutations	Restore WT p53 induction of apoptosis	In vivo p-53 deficient nude mouse xenograft	Higher survival rate compared to WT ad5 or ONYX-015 treatments
**CRAd, Ad5-Δ24-RGD** [96,130]	NA	CRAd, Ad5-Δ24-RGD	Rb and p53 mutations	Restore WT p53 induction of apoptosis	Phase I clinical trial NCT00562003	Future chemotherapy combination studies in development
p53-Bad* (2019) [104]	Gene Therapy	Ad	p53 mutations and WT p53	Restore WT p53 induction of apoptosis	In vitro	Induction of apoptosis regardless of p53 status
**Au-C225-p53** (2019) [73]	pCMV WT p53 Gene Therapy	Au NP/cetuximab	p53 mutations	Restore WT p53 induction of apoptosis	In vitro SKOV-3 cells and in vivo SKOV-3 xenograft mice	Positive; future clinical trials possible
**ZeFe-4 NPs** (2020) [76]	NA	ZeFe-4 NPs	Gain-of-function p53 mutations, loss of Zn(II) binding; R175H and R273H	Degrade gain-of-function p53 mutations	In vitro p53 S241F ES-2 ovarian cancer cells; In vivo orthotopically implanted p53 Y220C xenograft breast cancer model	Positive; minimal toxicity
**SPIO-Serum Nanoworms** (2017) [131]	NA	SPIO-serum Nanoworms	Overexpressed mutated p53	Induce ferroptosis	In vitro	Unknown
**Zinc Oxide NPs** (2017) [75,131]	NA	ZnO NPs	p53 mutations	Induce apoptosis via size-dependent cytotoxicity	In vitro SKOV-3 cells	Unknown
**ADGN-531-p53-mRNA** (2022) [132]	Gene Therapy	Nanocarrier ADGN-531-p53-mRNA	p53 mutations	Restore WT p53 induction of apoptosis. Restore PAPR inhibitor sensitivity	In vitro and in vivo	Unknown
**DDC-p53 Liposomes** (2007) [70,92]	Gene Therapy	Cationic Liposomes: DDC-DOTAP-DOPE	p53 mutations	Restore WT p53 induction of apoptosis	In vitro OVCAR-3 cells In vivo nude mice	Unknown
**ReACp53** (2011) [79,133]	Peptide	NA	R175H R282W	Restore WT p53 induction of apoptosis by eliminating p53 aggregation	In vivo xenograft model; combination with carboplatin	Minimal toxicity; increased efficacy of carboplatin in some patients
**p53-SLP vaccine (p53 70:248)** (2012) [134,135,136,137,138]	Overlapping Synthetic Long Peptides	Vaccine	Gain-of-function p53 mutations; overexpression of p53	Restore WT p53 induction of apoptosis	Phase II clinical trial	No change in response to secondary chemotherapy; induced immune response; potent
**p53 vaccine** (2012) [134,135,136,137,138]	Short Peptide	Vaccine (WT p53: 264–272)	Gain-of-function p53 mutations; overexpression of p53	Restore WT p53 induction of apoptosis	Tested in combination with interleukin 2 (IL-2)	Immune response; potent; some high-grade adverse events due to IL-2
**Arsenic Trioxide** (2019) [139,140,141,142]	Small Molecule	NA	R175H R273H R248Q	Restore WT p53 induction of apoptosis by reactivating misfolded p53 mutants; increase efficacy of PARP inhibitors	Phase II clinical trial (NCT04489706)	Adverse myelosuppression
**COTI-2** (2019) [143,144,145,146]	Small Molecule (thio-semicarbazone)	NA	p53 misfolded mutations	Reactivate misfolded mutant p53	Phase I clinical trial NCT02433626	Safe and well tolerated
**Kevetrin** (2020), [147]	Small Molecule	NA	p53 mutations	Induce cell cycle arrest and apoptosis	Phase II clinical trialNCT03042702	Stable disease (n = 2)
**PRIMA-1** (2019) [32,148,149,150,151]	Small Molecule (can be combined with SHetA2)	NA	p53 mutations	p53 reactivation and refolding with induction of apoptosis; reduces aggregation	Phase I clinical trial NCT04928508	Currently recruiting; prevented ovarian cancer in 67% of mice
**Eprenetapop-t APR-246 (PRIMA-1^MET^)** (2016) [152,153,154,155]	Small Molecule (can be combined with carboplatin or mebendazole)	NA	Preferentially effective in CIP2A-deficient patients	Restore WT p53 folding and subsequent induction of apoptosis	>10 clinical trials 1. Phase II clinical trial NCT03268382 2. Phase Ib/II clinical trial NCT02098343	1. Disease control rate of 70%; 2. disease control rate of 75%
**Zinc Metallochap-rones** (2022) [153,156,157,158,159,160,161,162]	Small Molecule	NA	R175H p53	Restore zinc binding; restore induction of apoptosis	In vitro In vivo	No synergistic effects with chemotherapies; toxic off-target effects due to zinc
**PC14586** (2021-present) [46,163,164]	Small Molecule	NA-oral	Y220C p53	Stabilize p53 and restore cell cycle arrest	Phase I/II clinical trials NCT04585750	Preliminary results indicate good safety profile

## Data Availability

Not applicable.
